# Electrocatalytic CO_2_ Reduction: From Homogeneous Catalysts to Heterogeneous-Based Reticular Chemistry

**DOI:** 10.3390/molecules23112835

**Published:** 2018-11-01

**Authors:** Abdulhadi A. Al-Omari, Zain H. Yamani, Ha L. Nguyen

**Affiliations:** 1 Department of Chemical Engineering, King Fahd University of Petroleum and Minerals, Dhahran 31261, Saudi Arabia; azhadi@kfupm.edu.sa; 2 Center for Research Excellence in Nanotechnology (CENT), King Fahd University of Petroleum and Minerals, Dhahran 31261, Saudi Arabia; zhyamani@kfupm.edu.sa; 3 Vietnam National University-Hochiminh, Hochiminh City 721337, Vietnam

**Keywords:** electrocatalytic CO_2_ reduction, reticular chemistry, metal-organic frameworks, covalent organic frameworks, renewable energy

## Abstract

CO_2_, emitted mainly from fossil fuel combustion, is one of the major greenhouse gases. CO_2_ could be converted into more valuable chemical feedstocks including CO, HCOOH, HCHO, CH_3_OH, or CH_4_. To reduce CO_2_, catalysts were designed and their unique characteristics were utilized based on types of reaction processes, including catalytic hydrogenation, complex metal hydrides, photocatalysis, biological reduction, and electrochemical reduction. Indeed, the electroreduction method has received much consideration lately due to the simple operation, as well as environmentally friendly procedures that need to be optimized by both of the catalysts and the electrochemical process. In the past few decades, we have witnessed an explosion in development in materials science—especially in regards to the porous crystalline materials based on the strong covalent bond of the organic linkers containing light elements (Covalent organic frameworks, COFs), as well as the hybrid materials that possess organic backbones and inorganic metal-oxo clusters (Metal-organic frameworks, MOFs). Owing to the large surface area and high active site density that belong to these tailorable structures, MOFs and COFs can be applied to many practical applications, such as gas storage and separation, drug release, sensing, and catalysis. Beyond those applications, which have been abundantly studied since the 1990s, CO_2_ reduction catalyzed by reticular and extended structures of MOFs or COFs has been more recently turned to the next step of state-of-the-art application. In this perspective, we highlight the achievement of homogeneous catalysts used for CO_2_ electrochemical conversion and contrast it with the advances in new porous catalyst-based reticular chemistry. We then discuss the role of new catalytic systems designed in light of reticular chemistry in the heterogeneous-catalyzed reduction of CO_2_.

## 1. Introduction

Carbon capture and storage (CCS) technologies have been developed in the past years due to the intensive demand for minimizing CO_2_ emissions, which come mainly from the combustion of fossil fuels—the primary cause of global warming [1]. This is now formally addressed amongst governments as witnessed by the Paris Agreement, which focuses on efforts to combat the increase in temperature and to adapt to the effects of CO_2_ emissions [2]. Sustainable techniques to alleviate the generation of high levels of CO_2_, attributed to the dramatic pace of industrial development, are numerous and include converting CO_2_ to chemical feedstocks or other valuable materials. This has been extensively explored and studied over the last few decades. Not only can these techniques reduce the high levels of CO_2_ emissions from industry, they may also provide a solution to fulfill the demand for renewable energy storage systems.

In order to convert CO_2_ into valuable chemicals or fuels, techniques including chemical fixation, hydrogenation, photocatalysis, and electrocatalysis are considered, depending on the nature of the catalyst [3,4,5,6]. However, it should be noted that CO_2_ is composed of the C=O bond, which is highly stable and lacking in reactivity. Hence, CO_2_ is chemically a highly stable molecule and requires high input energy to cleave the C=O bond [7]. Due to the obstacles of the C=O cleavage, as well as the high cost incurred by the process to capture CO_2_, the properties of the catalysts used for CO_2_ reduction have to be cleverly designed to meet the targets of large amounts of CO_2_ loading, high conversion efficiency, fewer by-products, and long catalyst lifetime. These prerequisites have turned the research in the direction of using photocatalysts and electrocatalysts for CO_2_ conversion due to their potential operation in practical applications and effectiveness in supplying energy to facilitate the chemical reduction processes.

Recently, the electrocatalytic CO_2_ reduction approach has emerged as a state-of-the-art topic in clean and renewable energy [8]. This approach requires electrocatalysts that are porous materials, such as metal organic frameworks (MOFs) or their sub-classes. These catalysts should possess a high internal surface area, which is useful for high CO_2_ uptake, and a high density of active catalytic sites for enhancing the energy conversion efficiency. There are many reviews detailing the scope of MOFs, as well as presenting the in-depth evaluation to comprehend the mechanisms and advances of using MOFs for electrocatalytic CO_2_ reduction [9,10,11], especially as MOFs are extended, highly porous, and crystalline materials that can be intentionally functionalized and whose structural features can be modified. In this perspective, we have avoided duplicating previous works. Instead, the advanced process of CO_2_ reduction by electrocatalysis will be presented to highlight the progress in this field from homogeneous to heterogeneous-based MOF materials and show the crucial role of designed porous materials (MOFs and sub-class) based on reticular chemistry.

## 2. Discussion

Numerous products can be produced by electrocatalytic CO_2_ reduction. This is due to the catalytic class and the operational condition of electrolytes leading to the reduction pathways due to two (2), four (4), six (6), or eight (8) electrons. Formed products include CO, formaldehyde or formic acid, methanol, or methane, respectively. It is noted that the process of CO_2_^•−^ generation from CO_2_ via single-electron reduction requires a potential of −1.90 V versus (vs.) the normal hydrogen electrode (NHE), which results from the large transformation energy necessary to reorganize the linear molecule to become a bent radical anion [12]. The proton-coupled multi-electron steps that occur during the reduction reaction are presented in Equations (1)–(6) and present the standard potentials of CO_2_ reduction in aqueous solutions. Given the fact that CO_2_ reduction catalyzed by an electrocatalyst will be conducted with a complicated mechanism, the chemical reduction is formed by multi-electron steps to produce a mixture of reduced products having various selectivity and reaction efficiencies. The high thermodynamic barrier and the competitive potential energy presented in the equations present challenges for the design and synthesis of an electrocatalyst that can meet the requirements of high selective performance as well as high reaction rates, in addition to stability.

CO_2_ + 2H^+^ + 2e^−^ → CO + H_2_O E° = −0.53 V (1)

CO_2_ + 2H^+^ + 2e^−^ → HCO_2_H E° = −0.61 V (2)

CO_2_ + 4H^+^ + 4e^−^ → HCHO + H_2_O E° = −0.48 V (3)

CO_2_ + 6H^+^ + 6e^−^ → CH_3_OH + H_2_O E° = −0.38 V (4)

CO_2_ + 8H^+^ + 8e^−^ → CH_4_ + 2H_2_O E° = −0.24 V (5)

CO_2_ + e^−^ → CO_2_^•−^ E° = −1.90 V (6)

### 2.1. Homogeneous Catalyst for CO_2_ Reduction

The homogeneous electrocatalyst can fulfill the role of an electron transfer reaction and facilitate the acceleration of a chemical reaction termed ‘chemical kinetic’, which, along with an electron transfer process, needs to be rapid for a promising homo-electrocatalyst. Two factors can be optimized via ligand design and the judicious selection of metal centers to adjust (i) the redox potential (E°) for the electron transfer and (ii) the certain chemical reaction (i.e., CO_2_ to CO) [13]. In the field of homogeneous catalysis for electrocatalytic CO_2_ reduction, metal complexes with macrocyclic ligands, metal complexes with bipyridine ligands, and transition metal phosphine complexes are the three main categories that have been well studied [14]. Due to the scope of this perspective, we will focus our attention on the reports of the category of homogeneous catalysts that have benchmarked results of faradaic efficiency (FE) and low overpotential.

In the 1970s, electrocatalysis of CO_2_ using cobalt and nickel phthalocyanines complexes was reported for the first time by Meshitsuka et al. [15], although without presenting clear results and turnover number (TON). However, this provided the fundamental probability and an understanding of using transition metal complexes for CO_2_ reduction by electrocatalysis. In 1980, Eisenberg and co-workers reported the structures of the tetraazomacrocyclic complexes of cobalt and nickel applied to the reduction of CO_2_ to CO at potentials ranging from 1.3 to 1.6 V vs. the saturated calomel electrode (SCE) with the nearly quantitative efficiency of 98% [16]. However, these complexes displayed low turnover frequencies (TOFs) in the range of 2 to 9 per hour. In order to identify the catalyst that can overcome the kinetic challenges of CO_2_ electroreduction, the ligands need to be well tuned and functionalized. Sauvage and co-workers reported that catalysts based on the Ni(II)-cyclam complex showed high-performance levels of stability and selectivity in CO_2_-to-CO reduction. The faradaic efficiency (FE) was found to be 96% at −0.86 V vs. SCE under aqueous conditions [17,18].

One of the most efficient homogeneous electrocatalysts developed by Savéant and co-workers is the class of Fe-based porphyrin complexes. In the early stage of this work, Fe(0) porphyrins were reported to be unstable, although they were able to catalyze the reduction of CO_2_ to CO at −1.8 V vs. SCE in *N*,*N*-dimethylformamide (DMF) solvent [19]. Their stability and reactivity were then improved by adding a Lewis acidic ion (Mg^2+^) [19]. The Fe(0) porphyrin system was then realized to improve its efficiency and extend its lifetime by adding the weak Brønsted acids (1-propanol, 2-pyrrolidine, or CF_3_CH_2_OH). The TON of this kind of catalyst was found to be 350 h^−1^ at −1.5 V vs. SCE, which is a high overpotential and is far from being practical [20].

**Scheme 1:**
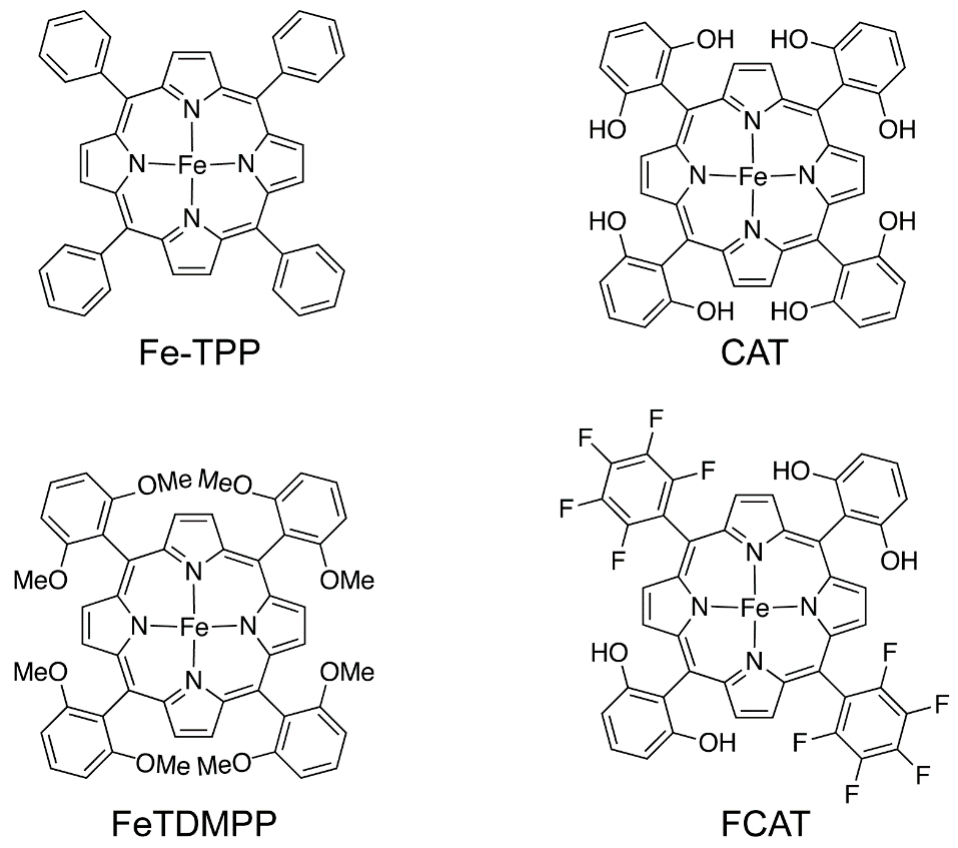
Iron-porphyrin-based complex catalysts for CO_2_ electrochemical reduction.

To reduce the overpotential and increase the TON of the CO_2_ reduction, porphyrin was further modified to functionalize the organic ligand forming a superior molecular system that can reduce CO_2_ to CO under a mixture of DMF and H_2_O [21]. Indeed, a local proton source was introduced by phenolic groups in all ortho and ortho′ positions of tetraphenylporphyrin (Scheme 1), enhancing the FE of CO_2_-to-CO reduction (above 90%). This electrocatalyst exhibited a very high TON of 50 million over 4 h of electrolysis at a low overpotential of 0.465 V. This kind of catalyst was further modified by adding both pendant acid groups and fluorine substituents [22] in the molecule to form a most efficient, electrogenerated Fe-porphyrin belonging to the benchmark of homogeneous catalysts for the CO_2_-to-CO conversion. In particular, Fe meso-tetra(2,6-dihydroxyphenyl)porphyrin (CAT) and Fe 5,15-di((2,6-dihydroxyphenyl)-10,20-di(penta uorophenyl)porphyrin (FCAT) were substituted for tetraphenylporphyrin (TPP) to construct the Fe-based complexes, which not only showed more affinity toward the CO_2_ molecules but presented the inter proton source providing the proton transfer from one of the local phenol groups. Fe-CAT and Fe-FCAT performed a very high FE of 100 ± 10% and 100 ± 5%, respectively, at a low overpotential (0.39 V for Fe-CAT and 0.45 V for Fe-FCAT).

### 2.2. Heterogeneous-Based MOFs Catalyst for CO_2_ Reduction

Although homogeneous catalysts perform the electrocatalytic CO_2_ conversion with a very high FE and selectivity at a low overpotential, their unrecyclability hinders the utilization of CO_2_ reduction in industrial applications. Additionally, forming multiple reduced products besides CO is still a challenge for homogeneous complex catalysts; they are weak on selectivity. On the contrary, heterogeneous electrocatalysts are able to overcome the afore-mentioned problem. Besides, the organic functional groups are intentionally decorated into the structure of a heterogeneous catalyst (i) to bring the suitable functionality at the proper step of the reaction process and (ii) to ideally design the “identified panel catalyst”, which contains the multivariate motifs for the multi-step activation of CO_2_ reduction [13]. Moreover, because (iii) the surface interaction between CO_2_ and catalyst and the mass transfer determines the reaction kinetics and current densities [23], a catalyst that has a high internal surface area permitting the high accessibility of CO_2_ could improve the performance of the electrocatalytic CO_2_ reduction. These characteristics lead to the quest for a material that is composed of a hybrid structure or extended and ordered network, which can be tailored to functionalize the desired organic active units—the generation of new porous and crystalline materials: MOFs [24] and COFs [11,25].

#### 2.2.1. Early State: Cu-MOFs and Open Metal Sites (OMS) Utilization

MOFs and COFs have been thoroughly investigated over the last few decades for their applications for gas storage (H_2_, CO_2_, and methane) and separation, catalytic transformation, and sensing [26]. However, the use of MOFs/COFs in electrochemical reduction has not matured yet. With respect to hydrocarbon forming, a Cu-based MOF was the first example that utilized a well-tuned framework to catalyze the reduction of CO_2_ under electrochemical conditions. Indeed, a Cu rubeanate metal-organic framework (CR-MOF) was successfully synthesized in 2012 by Hinogami et al. who placed a drop of CR-MOF on carbon paper (CP) to form a working electrode. CR-MOF was then exploited for its electronic characteristics including conductivity and dispersed reaction sites for electrocatalytic CO_2_ reduction producing HCOOH [27]. While the Cu metal electrodes catalyzed the CO_2_ electrochemical reaction to produce HCOOH, CO, and hydrocarbons (methane, ethylene, and ethane), CR-MOF catalyst produced HCOOH with an efficiency of 30% and a selectivity of 98%. This high selective electrocatalytic performance of CR-MOF was explained by the weak adsorption of CO_2_ on the reaction site of the ionic metal cluster in the structure of the CR-MOF. In particular, the CR-MOF electrode exhibited a high-quality product (HCOOH), which was 13-fold greater than that of the Cu metal electrode at −1.2 V vs. SCE. This result opened new insight into employing MOF for the electrochemical reduction of CO_2_.

At the same time, Kumar et al. demonstrated the use of Cu_3_(BTC)_2_ (HKUST-1), which was prepared as a uniform film in the electrochemical reduction of CO_2_ producing oxalic acid [28]. The cyclic voltammograms (CV) of Cu_3_(BTC)_2_ coated on a glassy carbon electrode displayed redox peak potentials of the reversible oxidation/reduction of Cu(II)/Cu(I) at −0.14 and +0.02 V vs. SCE, respectively. The reversible reduction/oxidation potential of Cu(0)/Cu(I) was found at −0.45 V and −0.102 V vs. SCE, respectively. An analysis of the products showed that oxalic acid was produced with an FE of 51% and a selectivity of 90%. In the pores of Cu_3_(BTC)_2_, CO_2_ intended to interact with the Cu-oxo cluster to generate the adducts, which were then stabilized by Cu(I) under CO_2_ saturated in DMF/tetrabutylammonium tetrafluoroborate solution. Those Cu(I) pieces, formed at potential −0.62 V vs. Ag/Ag+, catalyzed the reduction reaction. However, the pivotal role of the pore size and the interaction between CO_2_ molecules and the cluster of the catalyst were not discussed in detail. TON and the stability of the catalyst in the electrocatalytic CO_2_ reduction were also not reported in that work.

**Figure 1:**
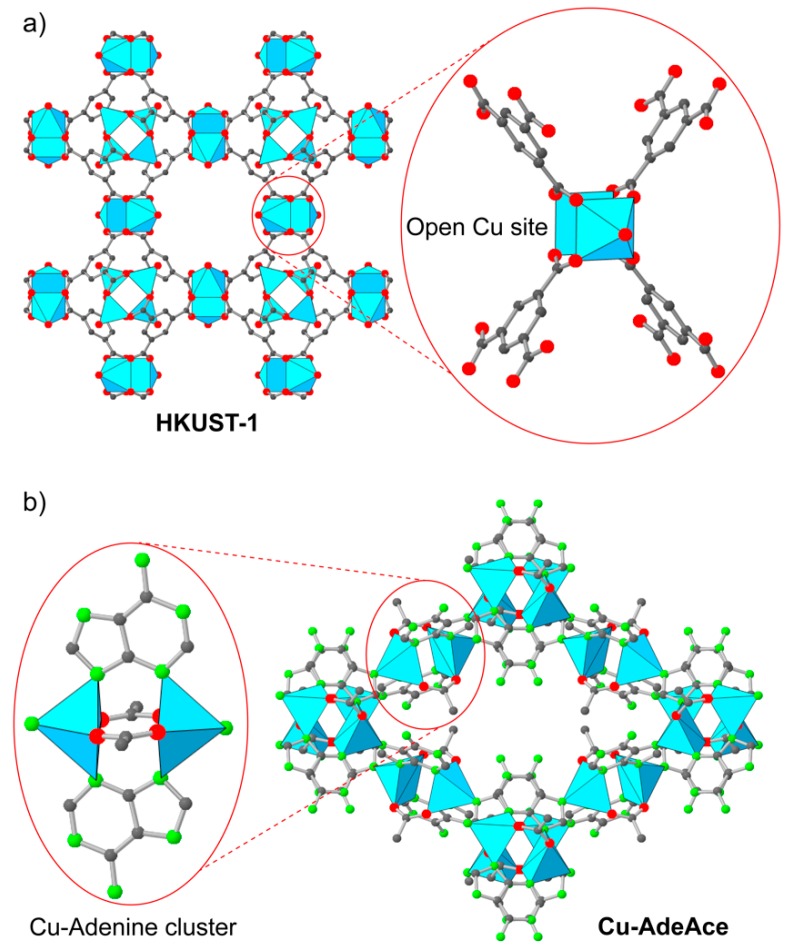
Crystal structure of Cu_3_(BTC)_2_ (HKUST-1) (**a**) showing the open metal site (OMS)-based cluster units and copper(II)–adeninate–acetate (Cu-AdeAce) (**b**).

Exploiting the opened Cu catalytic centers in MOFs, which have a relatively high surface area and accessibility (Figure 1), Albo and co-workers reported a study using HKUST-1 (Cu_3_(BTC)_2_), copper(II)–adeninate–acetate (CuAdeAce), Cu-organic aerogels (CuMOA) constructed from junctions and bis-bidentate dithiooxamidate (CuDTA), and CuZnDTA as favorable electrocatalysts for CO_2_ reduction [29] producing CH_3_OH and C_2_H_5_OH. In order to increase the surface area and the accessibility, as well as control the intrinsic pore system of MOFs, the above-mentioned MOFs were made as nanofibrous aerogels. MOF first formed a characteristic three-phase interface (gas–solid–liquid) by using the gas diffusion method. The resulting materials were subsequently investigated for the electrocatalytic reduction of CO_2_ displaying FE of 15.9, 1.2, 6.0, and 9.9% for HKUST-1, CuAdeAce, CuDTA, and CuZnDTA, respectively. In particular, HKUST-1- and CuZnDTA-based electrodes are the most active electrocatalysts for CO_2_ reduction, demonstrating working stability of up to 17 and 12 h, respectively.

This research indicated that the electrocatalytic performance is not only (i) correlated to the surface area of MOFs but is also (ii) dependent on the unsaturated coordination (Cu(II) pentacoordination of CuAdeAce hinders the electron accessibility by sterically surrounding the ligands). It should be noted that the open metal sites in MOFs could lead to the preferred electrochemical reduction of CO_2_ to alcohols, albeit the mechanism of the CO_2_-to-alcohol conversion was not fully evaluated [29].

#### 2.2.2. Functional Incorporation into MOFs

In the chemistry of MOFs, functional incorporation is a technique that allows the modification of the structural framework of the pristine materials to install novel functionalities without destroying or changing the structure [30]. It is noted that the functionalities in some cases cannot be incorporated into the structures by in situ synthesis owing to steric hindrance and kinetic challenges [30,31]. Instead, post-synthetic methods are applied to exchange/incorporate the targeted metal ion sources, organic linker-based functional groups, or metal nanoparticles (NPs) into the pores. The incorporation can be conducted through (i) in situ synthesis by using a mix and match strategy [32] or (ii) post-synthetic exchange [33].

**Figure 2:**
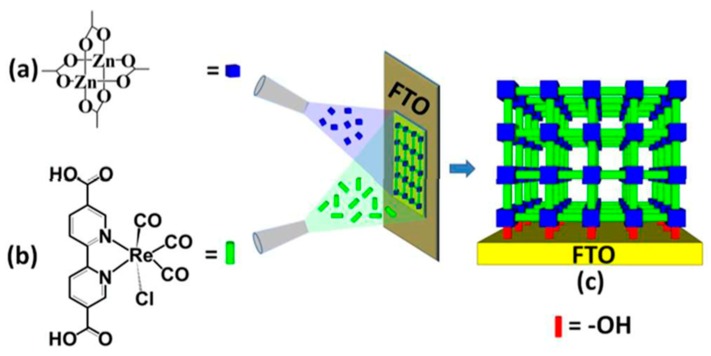
Fabrication procedure of Re-SURMOF constructed by (**a**) Zn-oxo cluster and (**b**) ReL(CO)_3_Cl (L = 2,2′-bipyridine-5,5′-dicarboxylic acid) linker on the conductive support (fluorine-doped tin oxide (FTO)). Reprinted with permission from ref. [33]. Copyright 2016 the Royal Society of Chemistry.

In 2016, Sun and co-workers reported a strategy to incorporate the catalytically active molecular complex-based carboxylate—ReL(CO)_3_Cl (L = 2,2′-bipyridine-5,5′-dicarboxylic acid)—into the structure of a highly oriented surface-grafted metal-organic framework (SURMOF) [33], which had already been investigated for the properties of charge transfer in the CO_2_ electrochemical reduction via liquid-phase epitaxy (LPE) method [34]. Re(CO)_3_Cl moieties contributed to the redox complex sites, catalyzing the CO_2_-to-CO conversion. The good organization of a layered structure of Re-SURMOF was deposited on the conductive fluorine-doped tin oxide (FTO) electrode through layer by layer growth with the support of a high-throughput automated spray system (Figure 2). Re-SURMOF network belongs to 2-D **sql** with preferred [001] orientation perpendicular to the FTO substrate, as confirmed by density functional theory (DFT) calculations. It exhibited a very high FE of 93 ± 5% towards CO_2_ reduction to CO. This result can be explained by the well-oriented layer sheets of Re-SURMOF that are favorable for charge transport from the electrode to the Re-based redox active sites. Only a small amount of H_2_ and no alcohols were found in the products. The TON was reported to be 580 after 2 h of electrocatalysis. The low stability of the Re-SURMOF was expected because of the degradation of Re(CO)_3_Cl complex. It was noted that for practical applications the stability of the electrocatalytic system needs to be improved.

**Figure 3:**
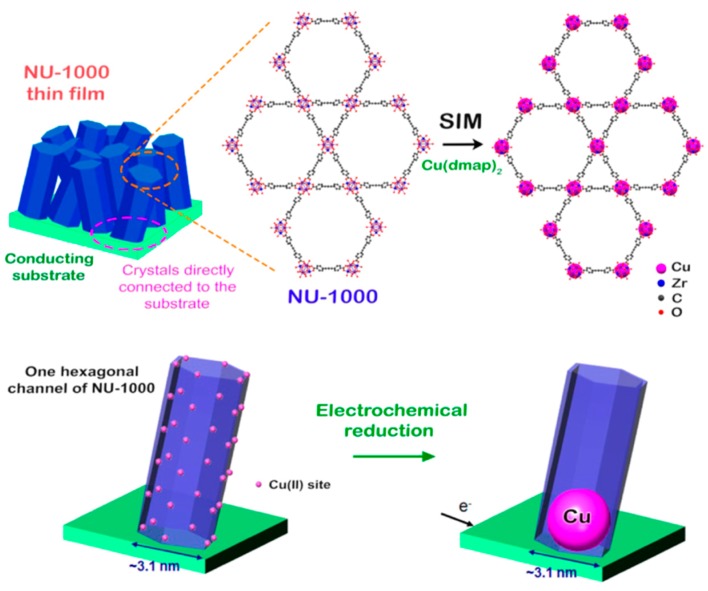
Crystal structure of NU-1000, which is metalated by Cu(dmap)_2_ by the solvothermal deposition in MOFs (SIM) technique. Cu(dmap)_2_ moieties surrounded within the channel of NU-1000 are electrochemically reduced to produce Cu nanoparticles (NPs). Reprinted with permission from ref. [35]. Copyright 2018 American Chemical Society.

Recently, by a combination of the stable porous MOF and the active metallic Cu nanoparticles (NPs) [35], Hupp, Farha and co-workers reported a MOF based on the tetra-carboxylate linker 1,3,6,8-tetrakis(*p*-benzoate)pyrene (TBAPy) and Zr_6_ node (Zr_6_(μ_3_-O)_4_(μ_3_-OH)_4_(OH)_4_(H_2_O)_4_) termed NU-1000, which was incorporated in Cu(dmap)_2_. This incorporation was carried out by the reaction between Cu^2+^ moieties and aquo and/or hydroxo ligands of the Zr-oxo cluster, via solvothermal deposition in MOFs (SIM) generating Cu-SIM NU-1000 (Figure 3). This material was then electrochemically reduced by heating in an H_2_ atmosphere and Cu(0) metallic NPs were subsequently formed due to movement and agglomeration. The resulting catalyst exhibited promising electrocatalytic performance for CO_2_ reduction in aqueous conditions. Proton nuclear magnetic resonance spectroscopy (^1^H-NMR) and gas chromatography (GC) detected the presence of a two-phase product: a liquid (HCOO^−^) and a gas phase (CO and H_2_). As the major product at −0.82 V vs. the reversible hydrogen electrode (RHE), a liquid-phase product (formate) was produced with an FE of 31%. It is noted that hydrogen evolution and electrochemical reduction occurred simultaneously during the reaction process, limiting the selective performance as well as the TON values (the estimated TON values for hydrogen, formate, and CO were 113, 47, and 4, respectively).

#### 2.2.3. Next Generation: Enhancing the Charge Carrier Mobility

By learning the lesson from homogeneous Fe-porphyrin utilization, a porphyrin-based linking unit can be considered to be spread out through the framework of MOFs for the following two scientific reasons: (i) exploiting the capabilities of MOFs for driving electron-transfer that is necessary for the electrocatalysis; (ii) introducing an active molecule into the structure of a catalyst acting as a conductive electrode whose features, including surface control, tunable pore cavities, and active site accessibility, along with permanent porosity and chemical stability, will be available for modification [36,37]. In 2015, Hod et al. utilized the post-synthetic approach to metalize MOF-525, a highly stable MOF constructed by [meso-tetra(4-carboxyphenyl)porphyrin] (TCPP) and trigonal prismatic Zr-oxo cluster, to build an efficient electrocatalytic system based on Fe-porphyrin linking units [37] with the hypothesis that the electrochemical CO_2_ conversion may be preferred through the hopping electron-transfer mechanism. The CO_2_ electroreduction result catalyzed by Fe_MOF-525 indicated that the Fe-metalated-based porphyrin MOF-525 was active for electrocatalytic CO_2_ reduction. In particular, CO_2_-to-CO conversion was found to be the main process with the nearly quantitative FE (~100%) at −1.3 V vs. NHE. However, the TOF was 16 times less than that of the homogeneous Fe-porphyrin complex [22,38] and the stability of the Fe_MOF-525 system was lower than that of other MOF-based electrocatalysts due to the catalytic degradation of Fe(II) active sites. This led to the pursuit of a new comprehensive catalyst that would not only tolerate the working operational condition of the electrochemical system but retain the electro-activity.

In order to develop the catalytically porous materials that allow the structural control (i.e., the functionality, catalytic metal center, and/or inorganic backbone), Yaghi and Yang and co-workers screened MOFs with systematically varied secondary building units (SBUs) and chose a cobalt–porphyrin MOF (Al_2_(OH)_2_TCPP-Co) for electrocatalytic reaction. This was due to the stability of Co-porphyrin units, which act as the active sites and can be maximized for CO_2_-to-CO electrochemical reduction [39]. The authors mentioned that the Co-porphyrin active sites in Al_2_(OH)_2_TCPP-Co were simultaneously exposed to the electrolyte—because of the layer-like framework of TCPP building units stacking to each other—and electrically connected to the transparent conductive fluorine-doped tin oxide (FTO) support via atomic layer deposition (ALD). The catalytic layer thickness could be further optimized for reactant diffusion and charge transport balancing. Particularly, the Al_2_(OH)_2_TCPP-Co thin layer promoted the electrocatalytic CO_2_ conversion producing CO and H_2_ as the two main products with the selectivity for CO of around 76% at −0.7 V vs. RHE. The TON of the reaction was estimated at around 1400 when assuming that all Co(II) atoms act as active sites. The catalytic stability of Al_2_(OH)_2_TCPP-Co was tested for over 7 h and showed no sign of a decrease in activity. 

**Figure 4:**
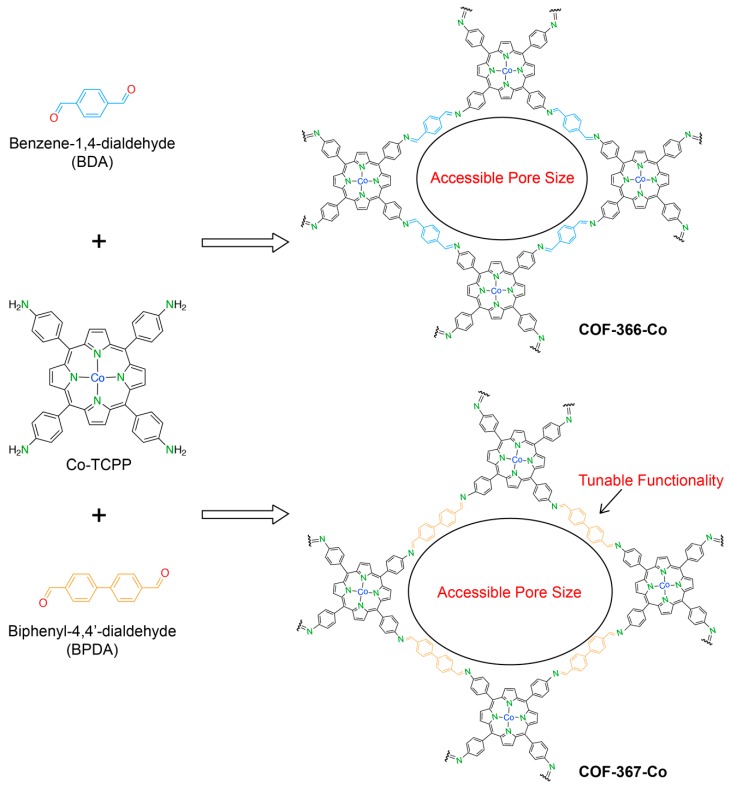
Schematic procedure for the synthesis of COF-366-Co and COF-367-Co—an isoreticular structure of COF-366-Co. Reticular chemistry is presented by tunable functionality and accessible pore structure.

By exploiting the advantages of crystalline porous materials, especially COFs, based on the design principles of reticular chemistry, the following features are precisely controlled: (i) the active molecular building unit can be predetermined to join into the frameworks; (ii) the post-synthetic method will be achieved to expand/functionalize the backbone without changing the topological network; (iii) the accessibility of the pores affecting the charge-transfer mechanism can be electronically tailored. These aforementioned properties of the electrocatalyst-based reticular system provide the opportunity to perform the multivariate character that can outperform the sum of the individual molecular parts [40]. With these notions, the utilization of covalent organic frameworks, COF-366-Co and COF-367-Co [40], comprised of Co-porphyrin-based linking units, which are linked to the extended structure by imine-bonding moieties for catalytic CO_2_ reduction by an electrochemical system, was reported by Lin et al. The crystal structure of COF-366-Co formed by the imine condensation reaction between 5,10,15,20-tetrakis(4-aminophenyl)porphinato]cobalt [Co(TAP)] and 1,4-benzenedicarboxaldehyde (BDA) (COF-367-Co is the isoreticular structure of COF-366-Co where the imine unit of COF-367-Co is a phenyl ring longer than that of COF-366-Co) revealed the π conjugation and π-π stacking system which is responsible for enhancing the charge carrier mobility (Figure 4). In particular, COF-366-Co exhibited the electrochemical reduction of CO_2_ to CO with an excellent FE of 90% at −0.67 V vs. NHE. The catalytic activity was stable for over 24 h and TON per electroactive cobalt was estimated to be 34,000 [40].

The longer version of COF-366-Co, COF-367-Co, which was obtained by reticular synthesis using biphenyl-4,4′-dicarboxaldehyde (BPDA) as a replacement for BDA, enlarged the pore accessibility, supporting the diffusion of reactants into the channel of COFs. The electrocatalytic result (FE of 91% and TON of 48,000) indicated that the expanded version of COF demonstrated improved catalytic properties compared to COF-366-Co. Additionally, the authors sought to investigate the effect of active Co sites regarding their catalytic performance. Indeed, bimetallic COF-367 derivatives, termed COF-367-Co(X%) (X = 1, 10 represents the percentage of Co-porphyrin active sites after introducing Cu sites) were chosen to conduct the CO_2_-to-CO electroreduction, leading to FE = 70% for COF-367-Co(10%) and FE = 40% for COF-367-Co(1%), which also displayed the highest TON of 296,000. This is due to the moderate percentage of active Co sites that are optimal for CO_2_ conversion over the proton reduction ability [40].

## 3. Conclusions and Outlook

In this perspective, the progress of electrocatalytic systems from homogeneous complexes to highly ordered and coordinated structures based on MOFs/COFs was highlighted and the process by which the electrochemical catalysts work and improve performance was discussed. Taking into account the exponential increase in research on porous materials—especially for the materials based on reticular chemistry, MOFs and COFs—the utilization of MOFs/COFs for electrocatalytic reduction has provided new insight into the field of materials science as promising candidates for electrochemical conversion of CO_2_.

In order to improve the performance of MOFs in the electrocatalytic reduction of CO_2_, there are many key factors that need to be controlled. (1) The interface between thin film MOFs and the conductive support electrode (i.e., FTO) has to be well stacked, thereby enhancing the adhesion between the MOFs and the electrode and facilitating the charge transfer. (2) The charge transfer can also be affected by controlling the structural frameworks, in which the catalyst containing layer-by-layer π stacking may be considered to be the potential catalytic system. (3) The high level of catalytic performance for CO_2_ electrochemical reduction also depends on the conductive properties, which are controlled by functionalities through the organic functional groups and/or metalation processes. (4) Multivariate components that include the possibilities of framework modifications, various functional groups, pore arrangements, surface accessibility, and active site enhancement [41], can be pre-designed to meet the requirements of the electrochemical reduction of CO_2_ and utilize synergistic phenomenon.

It is clear that MOFs/COFs display the advanced characteristics of porous and crystalline materials, which make them suitable for applications in renewable energy. MOFs/COFs are going to address the selectivity of CO_2_ reduction due to their outstanding features including their high CO_2_ affinity, large surface accessibility, and the confined pore space and well-defined active sites. However, one of the drawbacks for MOFs/COFs is the relevance of recyclability and activity. Still, under certain working condition, MOFs/COFs are cleverly designed to satisfy the requirement of retaining the active performance during the recycling, which will further increase the opportunity for using MOFs/COFs in practical applications. These prospective features will be responsible for the improved catalytic systems for CO_2_ reduction, which are precisely screened and designed utilizing the magic of reticular chemistry.
